# Role of DNA methylation in perinatal nicotine-induced development of heart ischemia-sensitive phenotype in rat offspring

**DOI:** 10.18632/oncotarget.20172

**Published:** 2017-08-10

**Authors:** Jun Ke, Nianguo Dong, Lei Wang, Yong Li, Chiranjib Dasgupta, Lubo Zhang, Daliao Xiao

**Affiliations:** ^1^ Department of Cardiovascular Surgery, Union Hospital, Tongji Medical College, Huazhong University of Science and Technology, Wuhan, China; ^2^ Department of Traditional Chinese Medicine, People's Hospital of Shanghai Putuo District, Shanghai, China; ^3^ Center for Perinatal Biology, Department of Basic Sciences, Loma Linda University School of Medicine, Loma Linda, California, USA

**Keywords:** perinatal nicotine, DNA methylation, heart ischemia-sensitive phenotype

## Abstract

**Background and purpose:**

Maternal cigarette smoking increases the risk of cardiovascular disease in offspring. Recently, we have demonstrated that perinatal nicotine exposure alters heart development and increases heart susceptibility to ischemia/reperfusion (I/R) injury in rat offspring. The present study tested the hypothesis that DNA methylation plays a key role in the nicotine-induced development of heart ischemia-sensitive phenotype in offspring.

**Experimental approach:**

Nicotine was administered to pregnant rats *via* subcutaneous osmotic minipumps from gestational day 4 until postnatal day 10. After birth, the postnatal offspring were treated with the DNA methylation inhibitor, 5-aza-2’-deoxycytidine (5-Aza) or saline from postnatal day 3 to day 10. Experiments were conducted in 1 month old offspring.

**Key results:**

Perinatal nicotine increased I/R-induced left ventricular (LV) injury, and decreased post-ischemic recovery of the LV function and coronary flow rate in both male and female offspring. Nicotine differentially increased DNMT3a expression and global DNA methylation levels in LV tissues. Treatment with 5-Aza inhibited nicotine-induced an increase in DNMT3a and global DNA methylation, and blocked the nicotine-induced increase in I/R injury and dysfunction in the heart. In addition, nicotine attenuated protein kinases C_ε_ and large-conductance Ca(2+)-activated K(+) (BKca) channel β1 subunit protein abundances in the heart, which were reversed by 5-Aza treatment.

**Conclusions and implications:**

The present findings provide novel evidence that the increased DNA methylation plays a causal role in nicotine-induced development of heart ischemic sensitive phenotype, and suggest a potential therapeutic target of DNA demethylation for the fetal programming of heart ischemic disease later in life.

## INTRODUCTION

Growing evidence suggests a key role of intrauterine adverse environment in determining the risk of disease in postnatal life. Maternal cigarette smoking during gestation is one of the most common causes of fetal growth restriction and a major risk factor in the development of cardiovascular disease in offspring [1, 2]. As being one of the key components in cigarette smoking, nicotine may contribute to the development of cardiovascular disorders [3]. Indeed, we and others have demonstrated in different animal models that fetal nicotine exposure causes fetal programming of cardiovascular dysfunction later in life [4–8]. Previous studies have reported that nicotine exposure, either from cigarette smoking or electronic cigarette (e-cigarette) can cause cardiac development defects [9]. Furthermore, our previous studies have shown an aberrant programmed cardiovascular function and development of heart ischemia-sensitive phenotype in response to intrauterine nicotine exposure [8, 10, 11]. However, the molecular epigenetic mechanisms underlying the perinatal nicotine-induced development of heart ischemia-sensitive phenotypes are not fully understood.

DNA methylation is one of the key epigenetic mechanisms in modification of gene expression patterns, and occurs at cytosine of the dinucleotide sequence CpG [12, 13]. Methylation in promoter regions is generally associated with repression of transcription, leading to a long-term shutdown of the associated gene. In contrast, hypo-methylation will result in increased gene expression. Clinical studies have shown that children exposed to maternal smoking demonstrate differences in global and gene-specific DNA methylation profiles when compared to their intact counterparts [14]. Of interest, our previous studies have also demonstrated that antenatal nicotine exposure causes alterations of specific gene expression patterns of cardiovascular tissues in both fetal and adult offspring. This is associated with aberrant methylation profiles of specific transcription factor binding sites at the gene promoter in a gender-dependent manner [10]. Of importance, modification of DNA methylation with a donor or inhibitor can epigenetically change the gene expression patterns and affect intrauterine programming of growth and disease throughout life [15, 16]. In addition, we have also demonstrated that the inhibition of DNA methylation can prevent norepinephrine-induced heart hypertrophy and increase heart ischemic injury [17]. These studies suggest that aberrant DNA methylation may contribute to the nicotine-mediated fetal programming of heart ischemia-sensitive phenotypes in postnatal life, and manipulation of the aberrant DNA methylation may recuse the adverse effect.

The present study was designed to reveal the potential epigenetic molecular mechanistic links between perinatal nicotine exposure and the aberrant development of heart ischemia-sensitive phenotype in offspring. We hypothesized that the nicotine-mediated aberrant DNA methylation profiles play a causal role in the development of heart ischemia-sensitive phenotype in offspring. Here, we presented direct evidence that perinatal nicotine exposure evokes global DNA hypermethylation in the heart of rat offspring, which leads to the reprogram of ischemia-associated protein expression patterns. This resulted in the development of heart ischemia-sensitive phenotype in postnatal life, whereas the inhibition of DNA methylation via a demethylation agent, 5-Aza, can effectively reverse severe pathological processes in rat offspring.

## RESULTS

### Inbibition of DNA methylation via 5-Aza reversed perinatal nicotine-induced LV dysfunction in offspring

**Figure 1:**
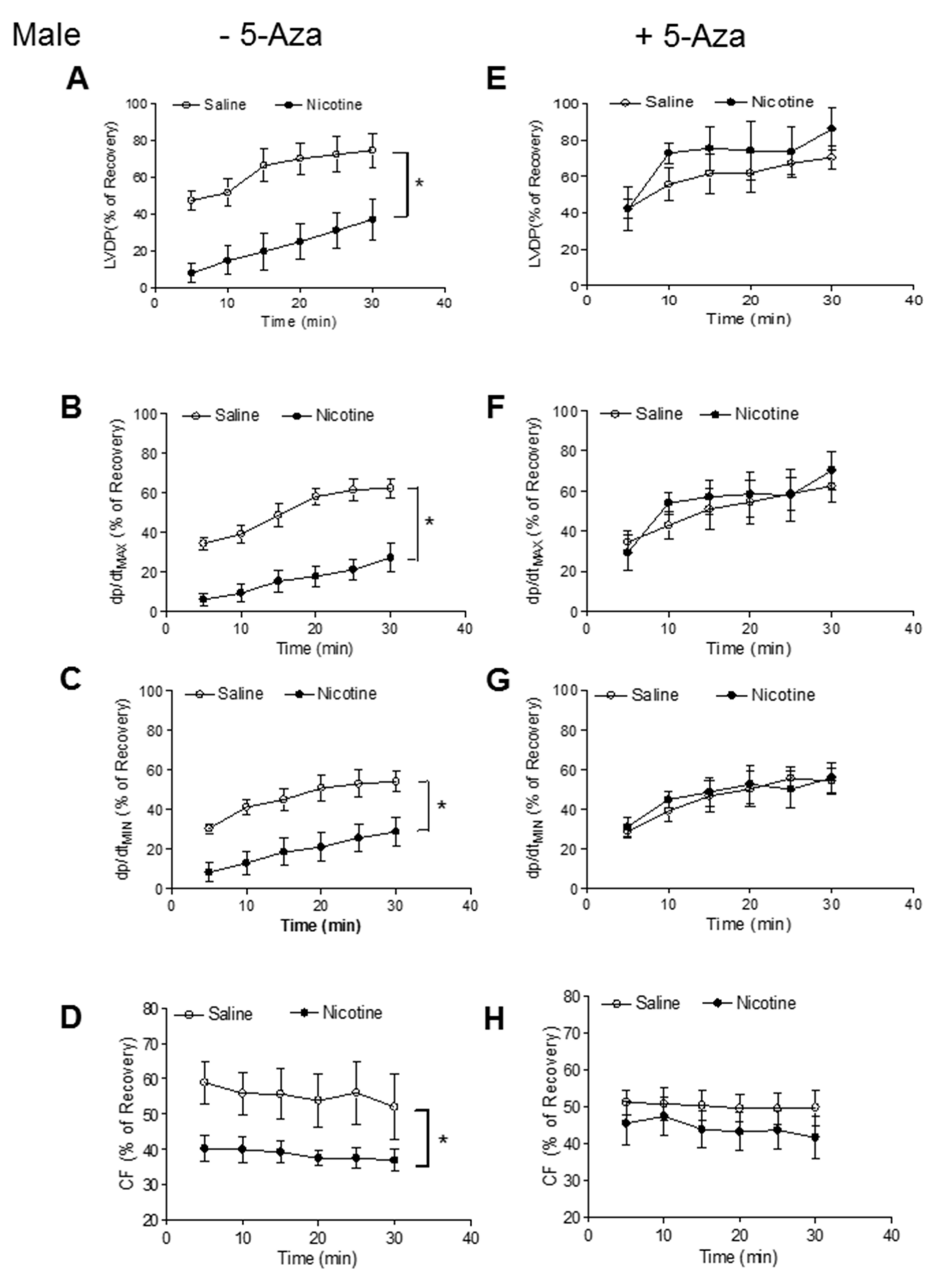
5-Aza reversed perinatal nicotine-induced decrease in post-ischemic recovery of LV function in male offspring Pregnant rats were treated with saline (control) or nicotine, and pups were administered with 5-Aza (i.p. 1mg/kg/day) on postnatal day 3, 7 and 10 after delivered. At one month of age, LVDP **(A, E)**, dp/dt_max_ **(B, F)**, dp/dt_min_ **(C, G)**, CF **(D, H)** of isolated hearts were determined after subjected to 30 min of ischemia and 30 min of reperfusion in Langendorff apparatus. Data are means ± SEM (n = 5-7 animals/group from different litter). * *P* < 0.05 *vs* control.

**Figure 2:**
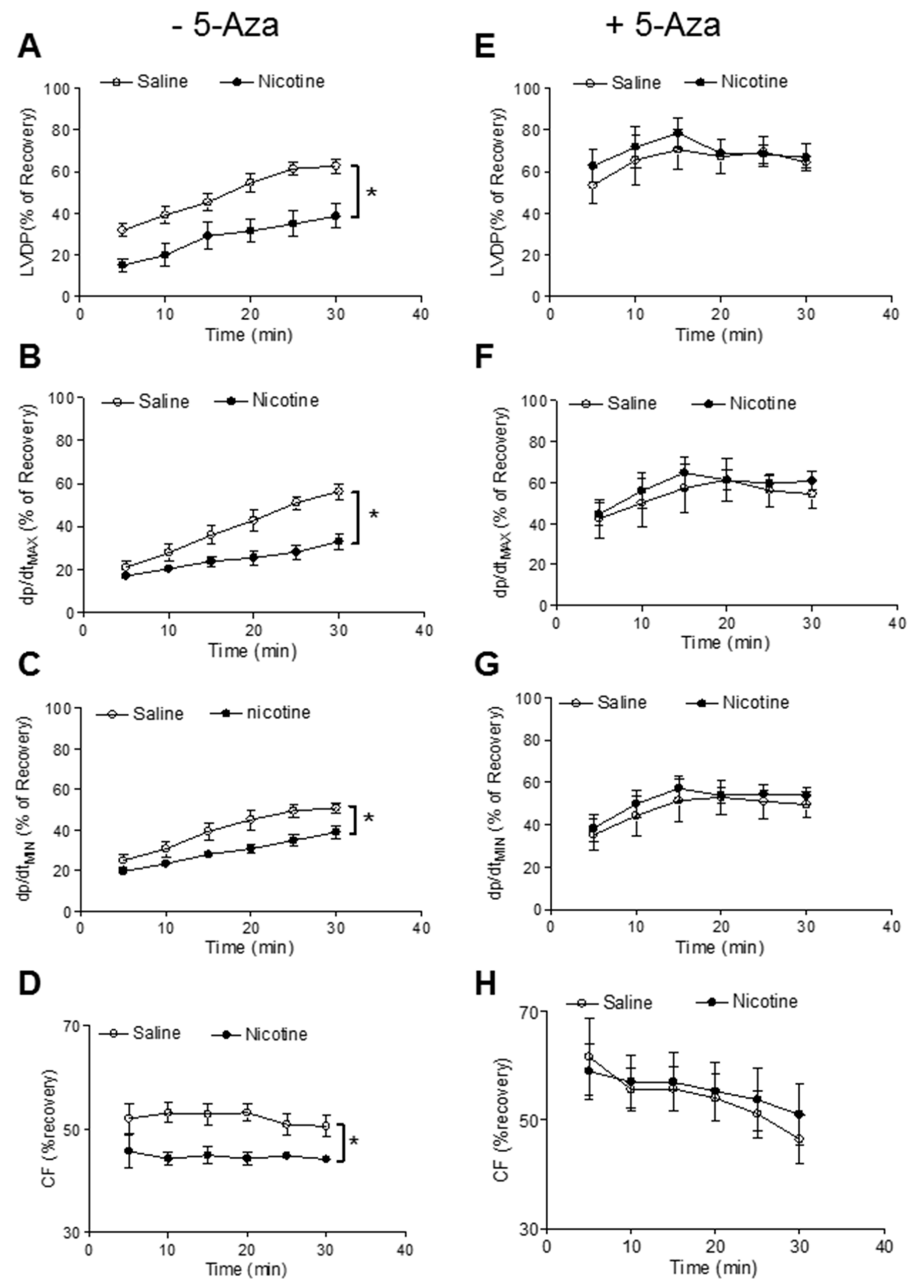
5-Aza reversed perinatal nicotine-induced decrease in post-ischemic recovery of LV function in female offspring Pregnant rats were treated with saline (control) or nicotine, and pups were administered with 5-Aza (i.p. 1mg/kg/day) on postnatal day 3, 7 and 10 after delivered. At one month of age, LVDP **(A, E)**, dp/dt_max_ **(B, F)**, dp/dt_min_ **(C, G)**, CF **(D, H)** of isolated hearts were determined after subjected to 30 min of ischemia and 30 min of reperfusion in Langendorff apparatus. Data are means ± SEM (n = 5-6 animals/group from different litter). * *P* < 0.05 *vs* control.

**Figure 3:**
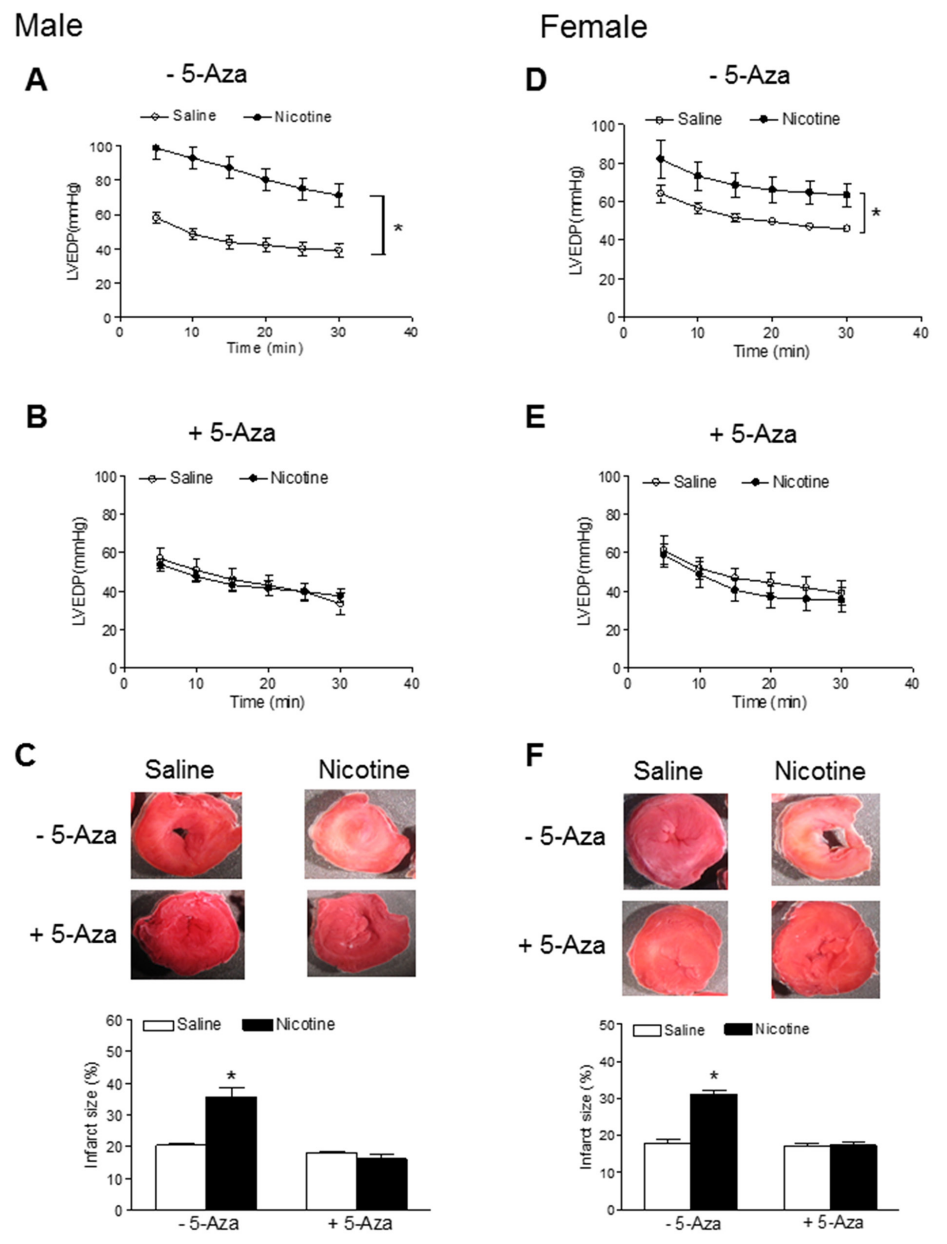
5-Aza prevented perinatal nicotine-induced increase in myocardial infarction in offspring Pregnant rats were treated with saline (control) or nicotine, and pups were administered with 5-Aza (i.p. 1mg/kg/day) on postnatal day 3, 7 and 10 after delivered. At one month of age, LVEDP of isolated hearts in the absence **(A, D)** or presence **(B, E)** of 5-Aza were determined after subjected to 30 min of ischemia and 30 min of reperfusion in Langendorff apparatus, and infarct size **(C, F)** of isolated hearts was determined by TTC staining. Data are means ± SEM (n = 5-6 animals/group from different litter). * *P* < 0.05 *vs* control.

Our previous studies have shown that perinatal nicotine exposure causes an aberrant programmed cardiovascular function and development of heart ischemia-sensitive phenotype in offspring [8, 10, 11]. Our previous studies have also demonstrated that nicotine exposure causes alterations of specific gene expression patterns of cardiovascular tissues, which is associated with aberrant methylation profiles at the gene promoter [10]. To elucidate the causal role of the DNA methylation on nicotine-induced adverse programming effects in hearts of offspring, we employed a 5-Aza to determine whether the inhibition of DNA methylation can prevent the perinatal nicotine exposure induced adverse functional outcomes in a heart I/R model *ex vivo* on rat offspring. Without the 5-Aza treatment, perinatal nicotine exposure significantly decreased the post-ischemic recovery of LVDP, dP/dt_max_, dP/dt_min_ and CF in both male (Figure 1) and female (Figure 2) offspring after 30 minutes of global ischemia. This was associated with increased LVEDP and myocardial infarct size in both the male and female offspring (Figure 3). However, treatment with 5-Aza, perinatal nicotine-mediated decreases in LVDP, dP/dt_max_, dP/dt_min_ and CF in both male (Figure 1) and female (Figure 2) offspring were abrogated. Furthermore, 5-Aza treatment also attenuated perinatal nicotine-mediated increases in LVEDP and myocardial infarct size in both male and female offspring (Figure 3). In addition, perinatal nicotine exposure had no significant effects on the baseline LV function in the 1-month-old rat offspring in the absence and presence of 5-Aza treatment (Table 1).

**Table 1:** Pre-ischemic Left Ventricular Functional Parameters

Animal group	HW/BW Ratio	LVDP	dP/dt_max_	dP/dt_min_	CF
	*g/g*	*mmHg*	*mmHg/s*	*mmHg/s*	*ml/min/g*
S_–Aza_ M (n=6)	0.005±0.0001	77±7	2709.8±151.8	1447.4±66.6	8.4±1.0
N_–Aza_ M (n=6)	0.005±0.0001	87.7±5.7	2879.5±163.2	1637.8±176.4	6.3±0.7
S_+Aza_ M (n=6)	0.006±0.0002	88.1±6.7	2606.5±147.4	1515.7±73.7	8.2±1.3
N_+Aza_ M (n=5)	0.007±0.0003	86.6±4.6	2596.4±124.4	1482.6±30.5	7.5±0.9
S_–Aza_ F (n=5)	0.006±0.0002	78.3±4.9	2751.8±106.7	1296.2±124.2	8.9±0.9
N_-Aza_ F (n=5)	0.005±0.0001	71.7±2.1	2247 ±104.20	1176.4±33.4	9.7±0.9
S_+Aza_ F (n=5)	0.006±0.0003	93.3±7.3	3043.4±414.6	1540±163.4	7.6±0.4
N_+Aza_ F (n=5)	0.006±0.0001	85.5±2.5	2789.5±197.7	1575±115.4	7.6±0.7

Note: HW, heart weight; BW, Body weight; LVDP, left ventricular developed pressure; dP/dtmax, maximal rate of contraction; dP/dtmin, maximal rate of relaxation; CF, coronary flow; S, saline; N, nicotine; Aza, 5-aza-2’-deoxycytidine; M, male; F, female.

### 5-Aza treatment abrogated the perinatal nicotine-induced an increase in the expression of DNMT3a and global DNA methylation levels in the LV tissues

**Figure 4:**
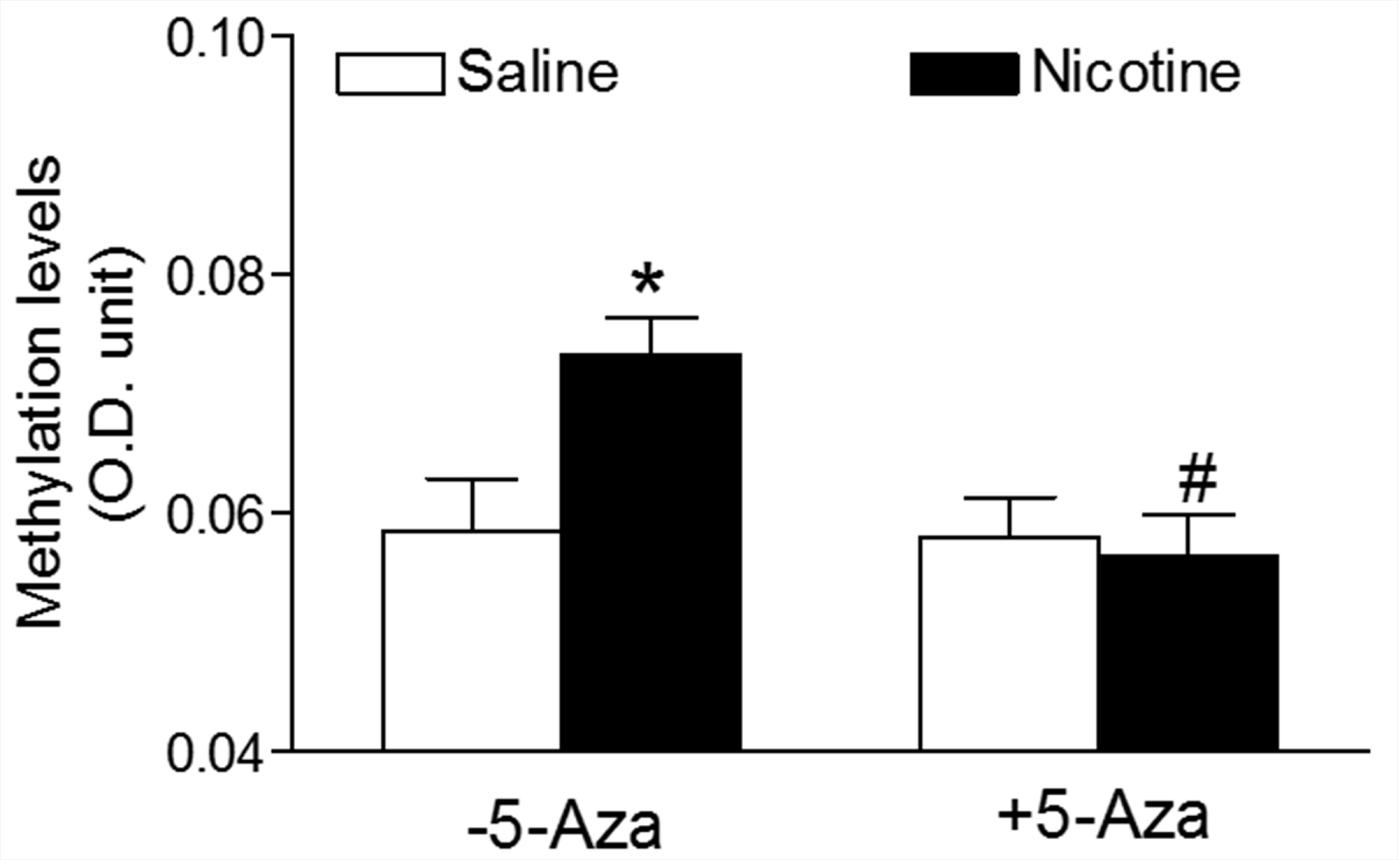
5-Aza abrogated perinatal nicotine-induced heightened global DNA methylation levels in the LV tissues of male offspring Pregnant rats were treated with saline (control) or nicotine, and pups were administered with 5-Aza (i.p. 1mg/kg/day) on postnatal day 3, 7 and 10 after delivered. At one month of age, the global DNA methylation was qualified by 5-mC ELISA in the left ventricle tissues isolated from male offspring. Data are means ± SEM (n = 4-6 animals/group from different litter). * *P* < 0.05 *vs* control. ^#^ P < 0.05 *vs* -5-Aza.

To reveal the causal roles of DNA methylation in nictoine exposure-induced adverse programming effects in offspring, we further evaluated the potential effects of perinatal nicotine exposure on global DNA methylation levels and the expression profiles of DNA methylation machinery in the heart of rat offspring. Because there were no gender differences in nicotine-mediated cardiac dysfunction (Figure 1–3), all of the following molecular biologic studies were only performed in male offspring. As shown in Figure 4, the global DNA methylation levels in the LV tissues of male rat offspring were significantly higher in the perinatal nicotine-treated group than those in the saline control group. 5-Aza treatment did not exert significant effects on the global methylation levels in the saline control groups, but significantly inhibited the DNA methylation in the perinatal nicotine-treated group and eliminated the differences between the saline control and nicotine-treated groups.

**Figure 5:**
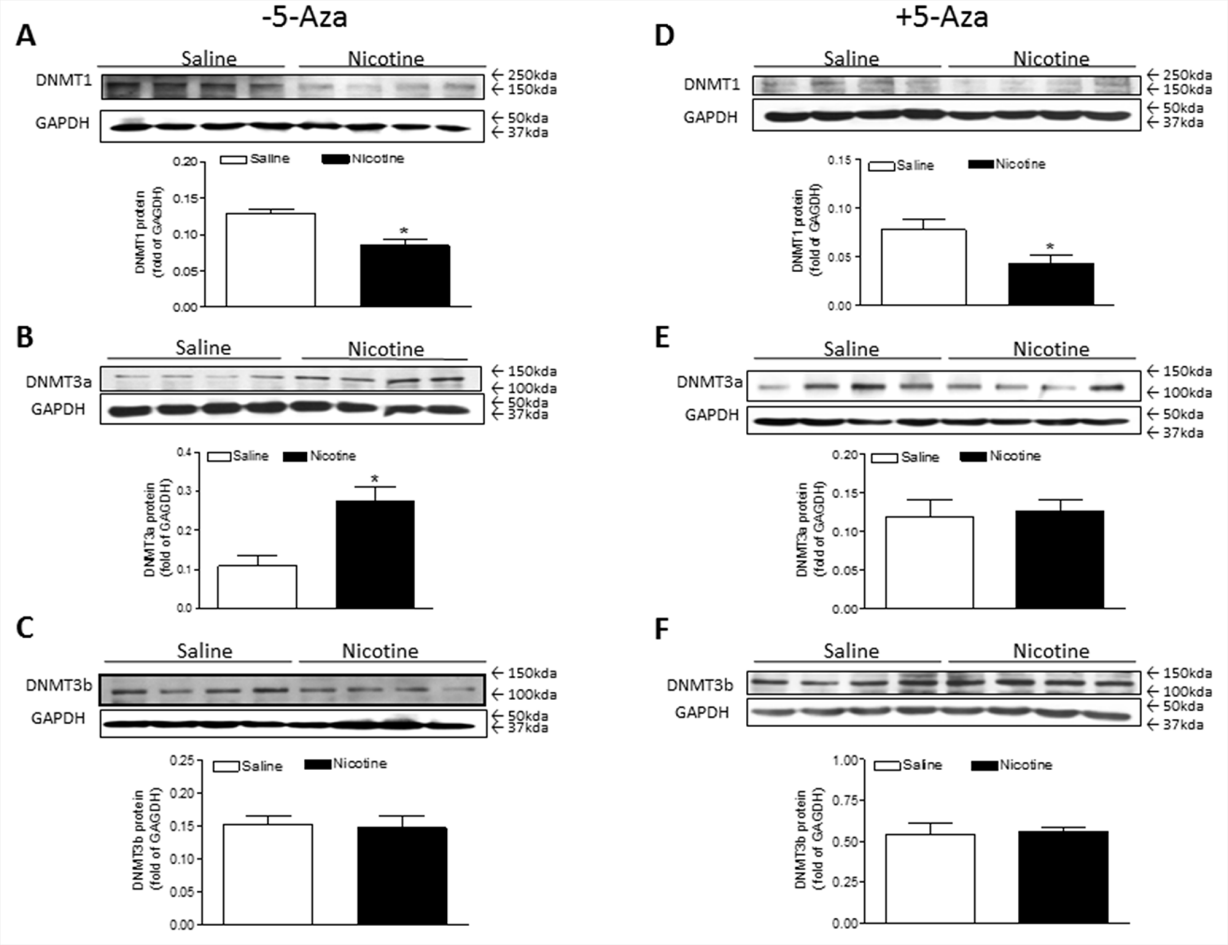
5-Aza restored perinatal nicotine-induced increase in the expression of DNMT3a in the LV tissues of male offspring Pregnant rats were treated with saline (control) or nicotine, and pups were administered with 5-Aza (i.p. 1mg/kg/day) on postnatal day 3, 7 and 10 after delivered. At one month of age, the protein abundances of DNMT1 **(A, D)**, DNMT3a **(B, E)**, and DNMT3b **(C, F)** were determined in the left ventricle tissues isolated from male offspring. Data are means ± SEM (n = 4 animals/group from different litter). * *P* < 0.05 *vs* control.

Interestingly, perinatal nicotine exposure conferred differential effects on the three major DNMTs gene expressions in the LV tissues of offspring. Perinatal nicotine exposure selectively reduced cardiac DNMT1 protein expression (Figure 5A) and enhanced the expression of DNMT3a (Figure 5B) but had no effects on the expression of DNMT3b (Figure 5C) as compared with the saline control groups. Treatment with 5-Aza had no effect on nicotine-mediated changes in cardiac DNMT1 protein expressions (Figure 5D), but significantly inhibited perinatal nictoine-mediated upregulation of DNMT3a (Figure 5E) in the cardiac tissues. In addition, the cardiac expression patterns of DNMT3b between saline control and nicotine-treated groups were not altered by the treatment of 5-Aza (Figure 5F).

### 5-Aza treatment restored the perinatal nicotine-induced repression of PKCε in the LV tissues

**Figure 6:**
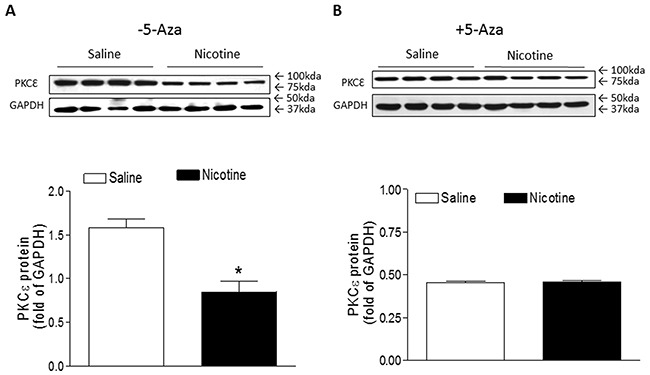
5-Aza restored perinatal nicotine-induced repression of PKCε in the LV tissues of male offspring Pregnant rats were treated with saline (control) or nicotine, and pups were administered with 5-Aza (i.p. 1mg/kg/day) on postnatal day 3, 7 and 10 after delivered. At one month of age, the protein abundance of PKCε in the absence **(A)** and in the presence **(B)** of 5-Aza treatment was determined in the left ventricle tissues isolated from male offspring. Data are means ± SEM (n = 4 animals/group from different litter). * *P* < 0.05 *vs* control.

Previous studies have confirmed that PKCε plays a pivotal role of cardioprotection in the cardiac ischemic injury. To see whether perinatal nicotine exposure alters cardiac PKCε expression patterns and whether inhibition of DNA methylation reverses nicotine-mediated changes of PKCε expression, we examined the cardiac PKCε expression levels in rat offspring in the absence of and presence of 5-Aza treatment. As shown in Figure 6A, perinatal nicotine exposure caused a significant decrease in the protein abundance of PKCε as compared with the saline control group in the absence of 5-Aza treatment. However, treatment with 5-Aza eliminated the differences of PKCε expression between the saline control and nicotine-treated groups (Figure 6B).

### 5-Aza treatment restored the perinatal nicotine-induced decrease in the expression of BKca channel β1 subunit in the LV tissues

**Figure 7:**
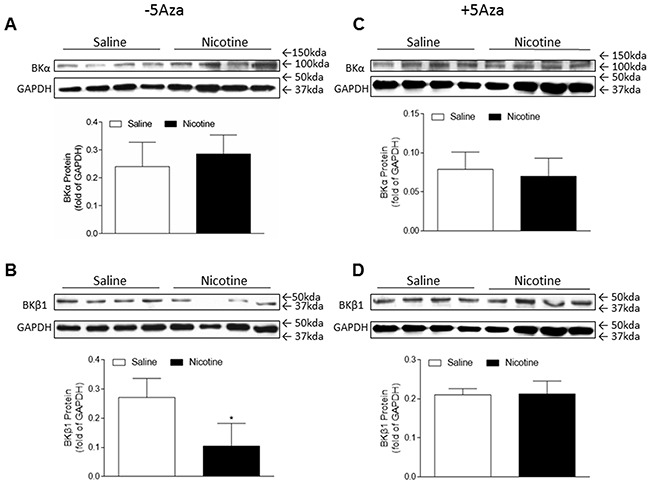
5-Aza abolished perinatal nicotine-induced decrease in the expression of BKca channel β1 subunit in the LV tissues of male offspring Pregnant rats were treated with saline (control) or nicotine, and pups were administered with 5-Aza (i.p. 1mg/kg/day) on postnatal day 3, 7 and 10 after delivered. At one month of age, the protein abundances of BKca channel β1 subunit **(B, D)** and α subunit **(A, C)** were determined in the left ventricle tissues isolated from male offspring. Data are means ± SEM (n = 4 animals/group from different litter). * *P* < 0.05 *vs* control.

**Figure 8:**
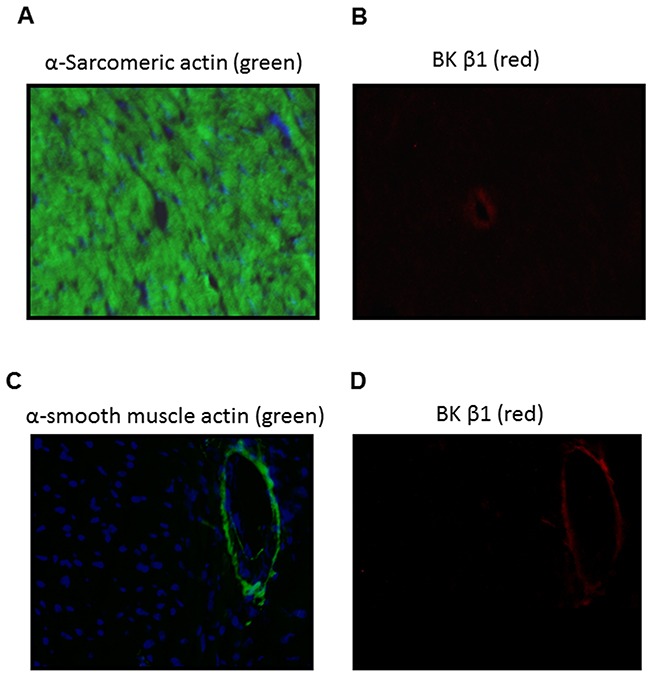
Localization of BKca channel β1 subunit in the LV tissues of male offspring Immunofluorescence staining of heart section with α-sarcomeric actin (**A**, green), α-smooth muscle actin (**C**, green), BKca channel β1 subunit (**B** and **D**, red). Nuclei were stained with DAPI (**A** and **C**, blue). BKca channel β1 subunit was detected in the coronary vascular walls **(B, D)**.

In the present study, our data (Figure 1) indicated that coronary flow rate was significantly reduced during the time course ischemia/reperfusion in the perinatal nicotine-treated group as compared with the saline control group. Given the fact that BKca channel is one of the key players involved in coronary vascular tone and heart reperfusion, we examined the effect of perinatal nicotine exposure on cardiac BKca channel expression patterns in the absence and presence of 5-Aza treatment. As shown in Figure 7, perinatal nicotine had no effect on the protein expression of BKca channel α subunit (Figure 7A), but significantly decreased the protein expression of BKca channel β1 subunit (Figure 7B) in the LV tissues as compared with the saline control group. However, in the treatment with 5-Aza, the protein expression levels of both BKcaα (Figure 7C) and BKca β1 (Figure 7D) subunits were not significant differences between nicotine-treated and saline control groups. In addition, immunofluorecsence staining in the LV tissues showed that BKca β1 subunit was predominantly localized in the coronary artery smooth muscle cells but not in the cardiomyocytes (Figure 8).

### Effects of perinatal nicotine exposure on caspase activities in the LV tissues

**Figure 9:**
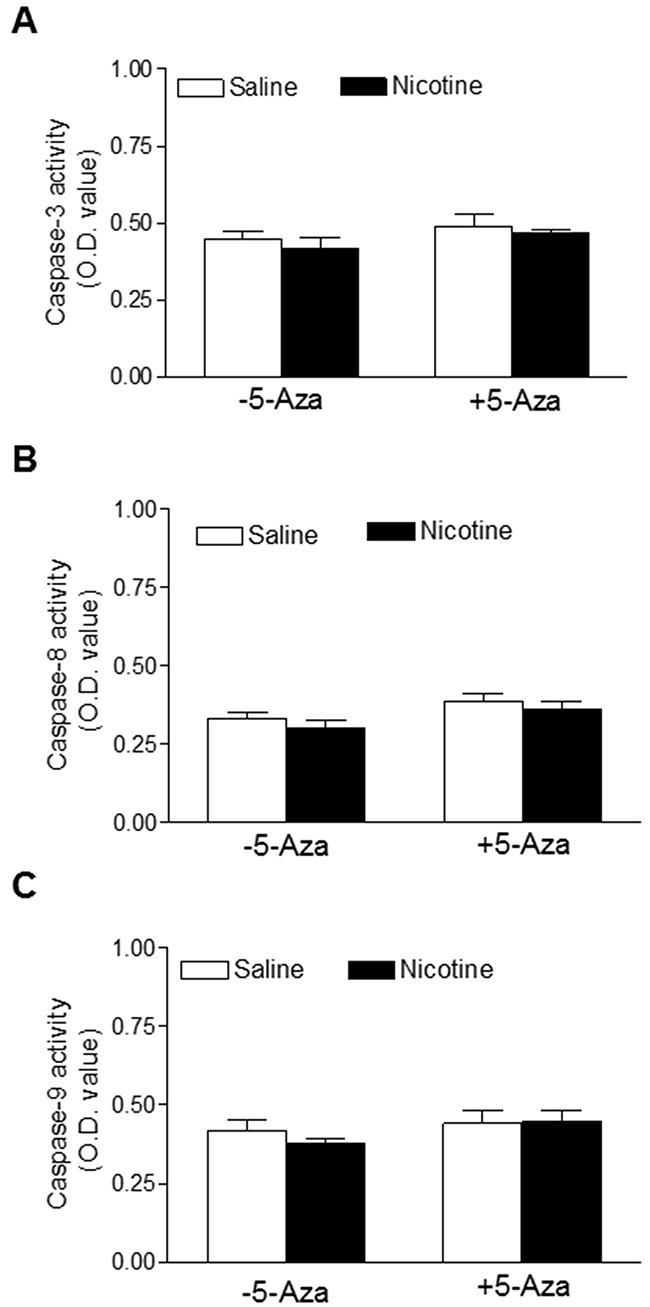
Effects of 5-Aza on perinatal nicotine-mediated changes of caspase activities in the LV tissues of male offspring Pregnant rats were treated with saline (control) or nicotine, and pups were administered with 5-Aza (i.p. 1mg/kg/day) on postnatal day 3, 7 and 10 after delivered. At one month of age, caspase activities **(A, B, C)** were determined in the left ventricle tissues isolated from male offspring. Data are means ± SEM (n = 4-7 animals/group from different litter). * *P* < 0.05 *vs* control.

Apoptosis is implicated in diverse pathological processes. The activation of caspase is a unique feature of apoptosis. Therefore, we examined the protease activities of caspase-3, caspase-8, and caspase-9 in the LV tissues. As shown in Figure 9, the activities of caspase-3, -8, or -9 activities in the LV tissues did not have significant differences between the nicotine-treated and saline control groups. In addition, the 5-Aza treatment had no effects on the caspase activities.

## DISCUSSION

Our previous studies have demonstrated that perinatal nicotine exposure alters fetal development and causes an aberrant development of cardiac ischemia-sensitive phenotype in offspring [8, 10, 11]. The present study provides further direct evidence that the alteration of DNA methylation is one of the key epigenetic molecular mechanisms underlying the nicotine-mediated development of heart ischemia-sensitive phenotype in offspring. There are two major findings in the present study: (1) perinatal nicotine exposure increased LV ischemia/reperfusion (I/R) injury and caused cardiac dysfunction, which was associated with an increase in DNA methylation and decreases in ischemia-related protein (such as PKCε and BKcaβ1) expressions; (2) inhibition of DNA methylation via a DNA-demethylation agent rescued the aberrant DNA methylation level, resulted in restoring the increased vulnerability to heart I/R injury in offspring. These findings clearly suggest a potentially novel therapeutic strategy of demethylation for treatment of perinatal nicotine-induced coronary heart ischemic disease.

Clinical studies have shown an alteration of global and gene-specific DNA methylation profiles in children exposed to maternal smoking [14]. Our previous studies in the animal model have also demonstrated that perinatal nicotine exposure causes alterations of specific gene expression patterns. This is associated with the aberrant methylation profiles of specific transcription factor binding sites at the gene promoter in cardiovascular and brain tissues [10, 18]. Consistent with the previous studies, the present study showed an increase in cardiac DNA methylation levels in offspring that had been perinatally exposed to nicotine. 5-Aza has been widely used as a DNA methylation inhibitor, which inhibits global and gene-specific DNA methylation [17–21]. In the present study, our data shows that treatment with 5-Aza has significantly inhibited nicotine-mediated increases in DNA methylation in cardiac tissues. This further suggests that 5-Aza can specifically inhibit DNA methylation in our animal model. Based on the important role of DNA methylation in gene regulation and normal development, injection newborn rat with too much or too early 5-Aza may affect pantosomatous organ functions. Indeed, our previous studies have shown that, when 5-Aza (1 μg/g/day) was administered to neonatal rats via intraperitoneal injection (*i.p.*) on postnatal day 1 to 3, this treatment significantly decreased the body weights and induced asymmetric growth restriction with the increased brain to body weight ratio and aberrant neurobehavioral functions in the rats [21]. In present study, we had slightly modified the administered time of 5-Aza on postnatal day 3 to10. Our current results indicated that treatment with 5-Aza on this time period did not affect the heart to body weight ratio and baseline heart functions in the normal rats (Table 1). These findings suggest that the effect of 5-Aza on neonatal development and organ function may depend on the administration time.

The current study indicates that perinatal nicotine exposure had no effect on pre-ischemic baseline values of cardiac function but enhanced the LV myocardiac infarct size and attenuated post-ischemic recovery of LV function after 30 minutes of global ischemia/reperfusion in one-month-old male and female offspring. This suggests that perinatal nicotine-mediated heart ischemic injury and dysfunction is in a gender-independent manner, which is consistent with previous studies showing that perinatal nicotine exposure increased cardiac I/R injury in 3 month-old male and female offspring [8]. However, numerous studies have well demonstrated a gender difference in fetal programming of cardiovascular disease in postnatal life. Our previous studies have also shown that perinatal nicotine exposure causes gender-dependent increases in vascular contractility, hypertensive responses, and brain ischemic injury in male but not female offspring [5, 6, 18]. These studies suggest that the gender different effects of perinatal nicotine may be tissue or organ dependent.

The present finding that perinatal nicotine exposure caused an aberrant development of heart ischemia-sensitive phenotype in postnatal life, suggests that epigenetic mechanisms are involved in fetal programming of cardiovasular disease late in life. There are at least three major epigenetic regulatory pathways including DNA methylation, histone modification and non-coding RNAs which are involved in reprogramming of the gene expression pattern and development of cardivascular disease. In this study, we found that treatment with 5-Aza significantly improved the I/R-induced LV injury and the post-ischemic recovery of heart function in perinatal nicotine-treated offspring. This observation provides direct evidence that nicotine-mediated DNA hypermethylation may be one of the key epigenetic molecular mechamisms underlying the fetal programming of heart ischemia-sensitive phenotypes later in life. DNA methylation levels are regulated and maintained by DNA methyltransferases (DNMTs). It has been well documented that DNMT1 is indispensable for embryonic development and maintaince of DNA methylation patterns. Whereas DNMT3a and DNMT3b function as a *de novo* DNA methylation during early embryogenesis and in the progress of disease [22–24]. Therefore, both DNMT1 and DNMT3a/3b are contributing to global level of DNA methylation in normal fetal and neonatal development. In the present study, we found that perinatal nicotine significantly increased global DNAmethylation level, which was associated a decresed DNMT1 and an increased DNMT3a protein expression in the LV tissues as compared with the saline control groups. This suggests that perinatal nicotine exposure can differentially regulate the DNMTs expressions in the heart. However, the increased DNMT3a expression may overcome the decreased DNMT1 expression, resulting in a net increased global level of DNA methylation in perinatal nicotine-treated offspring. Indeed, the finding that treatment with 5-Aza reversed the nicotine-mediated DNMT3a overexpression, further confirms that the DNMT3a overexpression may contribute to the cardiac DNA hypermethylation. The molecular mechanisms underlying the perinatal nicotine-induced alteration of DNMTs expression remain unclear. Previous studies have shown a key role of reactive oxygen species (ROS) in regulation of DNMTs expressions and DNA methylation patterns [25]. ROS-induced oxidative stress has a dual role in regulation of DNA methylation. ROS can induce DNA hypomethylation, but also can function as catalysts of DNA methylation, causing a specific gene promoter hypermethylation [25]. It suggests that ROS may differentially regulate DNMT1, 3a, 3b expressions. Given the fact that perinatal nicotine exposure enhanced cardiovascular ROS production in offspring [11, 26], we speculate that nicotine-mediated ROS may cause a differential regulation of DNMT1, 3a, and 3b expressions, resulting in alteration of DNA methylation patterns. It opens a wide door for us to further investigate the potential cause-consequence relationship between ROS and DNA methylation.

Our current finding that perinatal nicotine exposure significantly increased cardiac ischemia/reperfusion injury and heart dysfunction associated with a decrease in cardiac PKCε expression, suggests that PKCε repression may contribute to the nicotine-mediated increased cardiac I/R injury. Indeed, it has been well demonstrated that PKCε gene, an intrinsic cardio-protective protein, plays a crucial role of cardioprotection in heart I/R injury [27–30]. Our previous studies have also demonstrated that fetal stresses cause epigenetic down-regulation of cardiac PKCε expression later in life [10, 11, 31–33]. These studies suggest that down-regulation of cardiac PKCε expression may be one of the most common mechanisms in fetal stresses-induced development of heart ischemia-sensitive phenotype late in life. Although the molecular mechanisms underlying the perinatal nicotine-mediated down-regulation of cardiac PKCε expression remain unclear, our previous studies suggest that nicotine-mediated increased methylation at different specific transcription factor binding sites in the PKCε promoter may be one of the key mechanisms [10]. In this study, we found that treatment with 5-Aza eliminated the differences of PKCε expression levels between the nicotine-treated and saline control groups, which further validates the cause-consequence of DNA hypermethylation in epigenetic down-regulation of PKCε gene expression in response to perinatal nicotine exposure.

The current finding that the coronary flow rate during 30 minutes of I/R was significantly reduced in perinatal nicotine exposed offspring as compared with the saline control groups, suggests that nicotine-mediated I/R injury may not only dependent on the changes of intrinsic myocardio-protective genes (such as PKCε gene) but also be dependent on the changes of coronary contractile-related genes. BKca channel is one of the key gene proteins in regulating coronary vascular tone and coronary blood flow [34–36]. The BKca channel in vascular smooth muscle cells consists of the pore-forming α subunit and regulatory β1 subunit [35]. Activation of the channel induces hyperpolarization, leading to vascular relaxation. Decreased BKca β1 subunit expression has been observed in coronary heart ischemic disease and other cardiovascular disease in different animal models [37–39]. Consistent with those findings, our present studies showed that the protein levels of BKca β1 but not α subunit in LV tissues were significantly decreased in perinatal nicotine-treated offspring as compared with the saline control group. In addition, with the immunofluorescence technique we identified that BKca β1 predominantly localized in the coronary smooth muscle cells but not in the myocardiac cells. This data suggests that the decreased BKca β1 gene expression in coronary vasculatures may increase coronary vascular tone, resulting in decreased coronary artery reperfusion and enhanced heart ischemic injury in the perinatal nicotine exposed offspring. Furthermore, the present finding that treatment with 5-Aza reversed the nicotine-mediated down-regulation of BKca β1 gene expression, suggests that DNA hypermethylation may be one of the key epigenetic mechanisms underlying nicotine-mediated BKca β1 repression. Similar findings have been reported that gestational hypoxia-mediated hypermethylation at specific transcription factor binding sites in the BKca β1 gene promoter plays an important role in the down-regulation of BKca β1 protein expression and reduced BK_ca_ channel activity [40]. In our future studies, we will further investigate whether and how perinatal nicotine exposure alters the CpG methylation profiles at the promoter of BKca β1gene.

Apoptosis is well documented to be involved in diverse pathological processes. To further explore the underlying mechanisms in perinatal nicotine-induced heightened heart vulnerability to I/R injury, we examined the activities of some key apoptosis markers including caspase-3, caspase-8 and caspase-9 in the heart to see whether the apoptosis signaling pathway is involved in nicotine-mediated development of heart ischemia-sensitive phenotype in offspring. Our current data indicated that all of the caspase-3, caspase-8 and caspase-9 activities determined by the caspase activity assay kit were not significantly different between the saline control and nicotine treated animals in the absence or in the presence of the 5-Aza treatment. This is consistent with previous studies showing no differences of those three casapase protein levels which was determined by Western blot analysis between the saline control and nicotine-treated groups in the same animal model [8]. These findings suggest that the apoptosis signaling pathway may not be a major mechanism implicated in the perinatal nicotine-mediated development of heart ischemia-sensitive phenotype in rat offspring.

In conclusion, our present study provides novel evidence that aberrant DNA methylation is one of the key molecular linkers between perinatal nicotine exposure and the development of heart ischemia-sensitive phenotype later in life. Our data indicated that perinatal nicotine exposure selectivity enhanced DNMT3a expression, which may play a predominant role in nicotine-mediated DNA hypermethylation. The enhanced DNA methylation was associated with decreases in PKCε and BKca β1 protein expressions, resulting in the development of heart ischemia-sensitive phenotype later in life. Of importance, inhibition of DNA methylation via 5-Aza, reversed nicotine-mediated pathological effects and heart dysfunctions. These findings not only provide novel evidence to elucidate the molecular mechanisms underlying the perinatal nicotine-induced development of heart ischemia-sensitive phenotype but also suggest a potential therapeutic molecular target involving epigenetic modifications for fetal programming of heart ischemic disease.

## MATERIALS AND METHODS

### Experimental animals

All the procedures and protocols were approved by the Institutional Animal Care and Use Committee of Loma Linda University and followed the guidelines in the National Institutes of Health Guide for the Care and Use of Laboratory Animals. Time-dated (day 2 of gestation) pregnant Sprague-Dawley rats were purchased from Charles River Laboratories (Portage, MI) and housed individually in plexiglas acrylic plastic cages, located in air-conditioned rooms (room temperature 22°C, relative humidity 60%; lights on from 8:00 a.m. to 8:00 p.m.). Pellet food and tap water were available *ad libitum.* On day 4 of gestation, rats were anesthetized with isoflurane (5% for induction, 2% for maintenance) in oxygen (2L/min for induction, 1L/min for maintenance), and adequate anesthesia was determined by the loss of pedal withdrawal reflex and other reactions from the animals in response to pinching the toe, tail, or ear. Saline or nicotine (at 4ug/kg/min) were individually administered to pregnant rats through osmotic minipumps (type 2ML4, Alzet, Durect Corp., Cupertino, CA) and inserted under the skin on the back of rats until postnatal day 10, as described in detail previously [3, 5, 8]. A total of 17 pregnant rats were used in this project (n=9 for saline control, n=8 for nicotine treatment). All of the 9 saline-treated pregnant rats had given birth naturally and produced 86 pups (9.6 ±0.9 pups/litter). However, one of the 8 nicotine-treated pregnant rats had aborted and the other 7 pregnant rats had given birth naturally and produced 68 pups (9.7 ±0.3 pups/litter). After birth, the neonatal rats from each litter were randomly divided into four groups: 1) saline control - 5-Aza; 2) saline control + 5-Aza; 3) nicotine - 5-Aza; and 4) nicotine + 5-Aza. 5-Aza (1ug/g/day) or saline was respectively administered to neonatal rats via intraperitoneal (i.p.) injection on postnatal day 3, 7 and 10 as described previously [17, 41, 42]. Male and female rat offspring were sacrificed at one month of age, and the hearts were isolated from those treated groups for Western blot, ELISA, immunofluorescence staining and I/R functional studies.

### Heart subjected to ischemia and reperfusion

Rats were anesthetized with isoflurane (5% for induction, 2% for maintenance) in oxygen (2L/min for induction, 1L/min for maintenance), and adequate anesthesia was determined as described above. Then hearts were isolated and retrograde perfused via the aorta in a modified Langendorff apparatus, as previously described in detail [8, 43]. In brief, the constant perfusion pressure was 70 mmHg and the Krebs-Henseleit buffer containing 118.5mM NaCl, 4.7mM KCl, 1.2mM MgSO_4_, 1.2mM KH_2_PO_4_, 11mM glucose, 25mM NaHCO_3_ and 2mM CaCl_2_ was pre-warmed (37°C) and gassed with 95%O_2_ and 5%CO_2_. The left ventricular (LV) function was assessed by a Digi-Med Heart Performance Analyzer^TM^ through a pressure transducer connected to a saline-filled balloon inserted into the LV. LV end-diastolic pressure (LVEDP) was set at ~5mmHg. After the baseline recording, hearts were subjected to 30 min of global ischemia followed by 30 min of reperfusion. The left ventricle developed pressure (LVDP), dp/dt_max_, dp/dt_min_, LVEDP, heart rate (HR), and coronary flow (CF) were continuously recorded. At the end of reperfusion, the left ventricle tissues were collected for the following tests.

### Measurement of myocardial infarct size

At the end of reperfusion, LVs were collected and cut into four slices, incubated in 1% 2,3,5-triphenyltetrazolium chloride (TTC, Sigma-Aldrich) for 15 minutes at 37°C, and immersed in formalin for 60min. Each slice was then photographed separately, and the areas of myocardial infarction in each slice were analyzed by computerized planimetry (ImageJ, NIH), corrected for the tissue weight, summed for each heart, and expressed as a percentage of the total LV weight, as previously described [8, 43].

### Western blot analysis

Protein levels of DNA methyltransferase (DNMT) 1, 3a, 3b, protein kinase C eplison (PKCε), large-conductance Ca_2+_-activated K^+^ (BKca) subunit α and β1 in the LV tissues were determined with Western blot analysis. In brief, LV tissues isolated from the above 4 groups of male offspring were homogenized in a lysis buffer containing 150mM NaCl, 50mM Tris·HCl, 10mM EDTA, 0.1%Tween, 0.1% β-mercaptoethanol, 0.1mM phenylmethylsulfonyl fluoride, 5ug/ml leupeptin, and 5ug/ml aprotinin, pH7.4. Homogenates were then centrifuged at 4°C for 20 min at 10,000g, and supernatants were collected. Protein concentration was measured in supernatant using a protein assay kit from Bio-Rad. Equal quantity of proteins were loaded onto 7.5%-10% polyacrylamide gel with 0.1% sodium dodecyl sulfate and were separated by electrophoresis at 100 V for 90min. Proteins were then transferred onto nitrocellulose membranes. Nonspecific binding sites were blocked for 3 hours at room temperature in a Tris-buffered saline solution containing 5% non-fat dry milk. The membranes were then incubated with primary antibodies against DNMT1 (1:500, Cell Signaling Technology), DNMT3a (1:1000, Cell Signaling Technology), DNMT3b (1:1000, Novus Biologicals), PKCε (1:1000, Santa Cruz Biotechnology), BKca α (1:500, Santa Cruz Biotechnology) and BKca β1 (1:500, Santa Cruz Biotechnology) at 4°C, respectively. After washing, membranes were then incubated with secondary horseradish peroxidase-conjugated antibodies. Protein bands were visualized with enhanced chemiluminescence reagents, and blots were exposed to hyperfilm. The results were analyzed with the Kodak electrophoresis documentation analysis system and Kodak ID image analysis software. Band intensities were normalized to glyceraldehyde-3-phosphate dehydrogenase (GAPDH).

### Immunofluorescence staining and imaging

Fresh LV tissues were embedded in the optimal cutting temperature compound (OCT) on dry-ice and then stored at -80°C. Before staining, LV tissues were cut into 10 um thick slices on a microtome-cryostat and mounted on super-frost plus slides. The tissue slides were then warmed at room temperature for 30 minutes and fixed in ice cold acetone for 5 minutes and then dried in the air. The following primary antibodies were employed: mouse anti-α smooth muscle actin (Sigma-Aldrich), mouse anti-α-actin (sarcomeric, Sigma-Aldrich) and anti-rabbit BKca β1 (Santa Cruz). After blocking with 5% bovine serum albumin(BSA) for 1 hour at room temperature and incubation with the primary antibodies at 4°C overnight, tissue slides were then incubated with the secondary antibodies raised against mouse and rabbit IgG conjugated with FITC and Texas Red (Santa Cruz) respectively for 1 hour at room temperature. After rinsing in PBS-Tween20 for several times, the slides were counterstained with DAPI for 1 minute and rinsed in PBS-Tween20 several times. The slides were then covered with an anti-fade mounting medium and visualized using EVOS FLc (Fisher).

### Caspase activity assay

The activities of caspases-3, caspase-8 and caspase-9 in the LV tissues were assessed using a colorimetric caspase-3, caspase-8, and caspase-9 kit (BioVision Inc, CA) following the manufacturer's protocol. Briefly, fresh LV tissues were homogenized in a lysis buffer provided by the manufacturer. Homogenates were then centrifuged at 4°C for 3 min at 10,000g, and supernatants were collected. Protein concentration was measured in the supernatant using a protein assay kit from Bio-Rad. Then, 50ul of supernatant containing 200ug protein and 50ul reaction buffer were incubated with 5ul of caspase-specific colorimetric substrates (0.2mM), DEVD-ρNA (for caspase-3), IETD-ρNA (for caspase-8) or LEHD-ρNA (for caspase-9) respectively in 96-well plates at 37°C for 1 hour. The absorbance was measured at 405 nm with a microtiter plate reader. Absorbance of blank samples was used as a background.

### 5-mC DNA ELISA

Global DNA methylation level was determined by the detection of global 5-methylcytosine (5-mC) in genomic DNA that was isolated from the LV tissues using a 5-mC DNA ELISA kit (Zymo Research), according to the manufacturer's instructions. In brief, 100ng of genomic DNA isolated from the LV tissues and standard controls provided by the kit were denatured at 98°C for 5 minutes in a thermal cycler, and then added into the plate wells with 5-mC coating buffer. After incubation at 37°C for 1h, the wells were washed with 5-mC ELISA buffer 3 times and then an antibody mix consisting of anti-5-mC and a secondary antibody was added to each well. The plate was covered with foil and incubated at 37°C for 1h. Then wells were washed with 5-mC ELISA buffer 3 times and a HRP developer was added to each well and incubated at room temperature for 1 h. The absorbance at 405 nm was measured using an ELISA plate reader. The 5-mC percentage for DNA samples was calculated using the logarithmic second-order regression equation at the standard curve that was constructed with negative and positive controls in the same experiment.

### Statistical analysis

Data are expressed as mean ± SEM. Experimental number represents rats from different dams. Statistical significance (*P<0.05*) was determined by analysis of variance (ANOVA) followed by Newman-Keuls post hoc testing or by Student's *t* test, when appropriate.

## Abbreviations

5-Aza: 5-aza-2’-deoxycytidine
i.p.: intraperitoneal
LVDP: left ventricle developed pressure
LV: left ventricle
dp/dt_max_: maximal rate of the increase of left ventricular pressure
dp/dt_min_: maximal rate of the decrease of left ventricular pressure
LVEDP: left ventricular end-diastole pressure
CF: coronary flow
I/R: ischemia/reperfusion
DNMT: DNA methyltransferase
PKCε: protein kinase C eplison
BKca: large-conductance Ca_2+_-activated K^+^
ELISA: enzyme-linked immunosorbent assay
GAPDH: glyceraldehyde-3-phosphate dehydrogenase
BSA: bovine serum albumin
5-mC: 5-methylcytosine
ANOVA: analysis of variance
